# Use of Over-the-Counter Medication among Pregnant Women in Sharjah, United Arab Emirates

**DOI:** 10.1155/2017/4503793

**Published:** 2017-07-19

**Authors:** Abduelmula R. Abduelkarem, Hafsa Mustafa

**Affiliations:** ^1^College of Pharmacy, University of Sharjah, Sharjah, UAE; ^2^AME Global FZE, Sharjah, UAE

## Abstract

**Background:**

Over-the-counter medications are widely available in pharmacies Their safety profile, however, does not extend to pregnant women. Accordingly, there should be educational programs developed for pregnant women to protect them from the harms of the side effects.

**Aim:**

This study was planned and designed with the aim of exploring the awareness and assessing the usage of OTC medications among pregnant women in Sharjah, UAE.

**Method:**

A cross-sectional survey using a self-administered questionnaire.

**Results:**

More than three-quarters (75.7%) reported that they are familiar with the term “over-the-counter drugs.” Interestingly, 40% of the respondents reported that they took OTC drugs during pregnancy, and the majority (94.2%) agreed with the survey statement “not all OTC medications are safe to be taken during pregnancy.” Constipation was the most frequent side effect that most of the participants reported during the study period. Folic acid (36%), calcium (28.6%), and iron (35.1%) were the most common supplements used by the pregnant women responding.

**Conclusion:**

The reported 40% usage of OTC medications among pregnant women in this study is worrisome and calls for the need to educate, counsel, and increase awareness among pregnant women regarding the dangers of OTC drugs usage while pregnant in Sharjah, UAE.

## 1. Introduction

Self-medication is the treatment of common health problems with medicines designed and labeled for use without professional supervision and approved as safe and effective for such use, as defined by the World Self-Medication Industry. Over-the-counter (OTC) drugs have been widely used in self-medication, for many years, in the treatment of common pregnancy related health problems. Pregnancy is a dynamic process in which anatomic and physiological changes occur from fertilization to parturition. Any given over-the-counter agent has vastly different effects depending on the stage of embryo and fetal development [1]. Accordingly, when a pregnant female uses OTC products for self-medication, she is exposing herself and/or her baby to different toxicological effects brought about by these drugs. At any point in the gestation period, over 90% of pregnant women take a prescription or OTC medication [2]. The common symptoms of pregnancy such as nausea, vomiting, heartburn, backache, constipation, or even migraine, pains, and cough require medications that should be selected carefully to avoid side effects on the fetus and mother [3]. It is probably due to these symptoms that pregnant females are reaching out to OTC medications. Also, it has been demonstrated that the most common types of OTC medications used by pregnant women are allergy medications, analgesics, respiratory medications, gastrointestinal medications, and skin condition products [4–6].

The United Arab Emirates (UAE) is a federation of seven gulf emirates with an estimated population of 9 million people in 2016, with 80% of the population comprised of expatriates from different countries [7]. Despite being with an estimated GDP of $370 billion in 2016 and a rapidly increasing population, there is a paucity of published research regarding people living below the poverty level [8, 9]. This rapidly increasing population necessitates that pharmacists and other members of the healthcare teams have access to adequate and up-to-date information on OTC products and are able to provide advice on the benefits and risks of using such medications to consumers, including pregnant women. Currently, there is a scarcity of data about the practice and impact of OTC medication usage among pregnant women in UAE. Accordingly, this study was planned and designed with the aim of exploring the awareness and assessing the usage of OTC medications among pregnant women in Sharjah, UAE.

## 2. Method

### 2.1. Ethical Consideration

The study was conducted after the approval of the University of Sharjah Ethics Committee, Sharjah, UAE (reference number: REC-16-10-03-01-S).

### 2.2. Study Design

A cross-sectional survey was conducted to assess the level of awareness and knowledge of pregnant women concerning OTC drugs. The study took place in the Emirate of Sharjah, UAE, over a period of three months (October to December 2016).

### 2.3. Study Population

Sharjah is the third largest of the seven emirates that make up the UAE and is the only one to have land on both the Arabian Gulf Coast and the Gulf of Oman. Residents of Sharjah represent around 19% of the UAE's population (4.76 million) (Ministry of Economy, 2008) [10]. Within the UAE, it has been reported that the crude birth rate or birth rate per 1,000 population was 15.54 during the year of 2014. The reported birth rate declined to 10.59 in 2015 according to the World Bank collection of development indicators [11, 12].

This study was conducted at four different clinics/hospitals within Sharjah: Al Qassimi Hospital, Dr. Oras Medical Centre, Bait Al Seha Clinic, and Dr. Rabha Habib Al Sayegh Clinic. A total of 140 questionnaires were distributed among these locations as 140 pregnant patients (of varying trimesters) visited the clinic during the study period. All the women who visited the clinics during this period were invited to take part in the study; the response rate was 100%. If a woman was pregnant (at any trimester), she was eligible to participate in the study. The only other inclusion criterion for this study was the patient's ability to understand either English or Arabic. Every woman who agreed to participate in the study was informed that her name and her responses to the survey questions, as well as any statements, would be treated as confidential and would not be used for any other purpose, apart from the study and that only aggregated data would be published. As the women filled out the survey questions, the researchers were available to clarify any misinterpretation or answer questions the participants may have.

### 2.4. Questionnaire Development

A 19-item questionnaire (Appendix) was developed in both English and Arabic versions, using items from surveys from previous studies [13–15]. The Arabic questionnaire was read and modified by Arabic language experts in order to validate the accuracy from its English translation. The survey was modified and amended to fit the UAE society, and any outdated or unnecessary questions were eliminated. It should be emphasized that the development of the questionnaire in Arabic involved considerably more effort than just mere translation. Sometimes it was not possible to find an equivalent term in Arabic that expresses a medical term in English. Moreover, a direct translation of an English phrase might be meaningless in Arabic or, even worse, lead to misunderstandings, unless the phrase was elaborated on or further qualified. For this reason, the Arabic questionnaire was reviewed and modified by Arabic language experts to accurately reflect the meaning from the English version. Furthermore, the survey was not produced in other languages such as Urdu or Hindi, because, as mentioned in the study design and population description, pregnant women who were unable to speak/read English or Arabic were excluded from the study.

The survey was divided into six sections (a copy of the questionnaire is available from the authors). Section 1 comprised personal information (Q1–Q6). The aim of Section 1 was to assess the demographic data of the participants such as nationality, age, marital status, education level, employment situation, and parity. Level of knowledge (Q7–Q10) was assessed in Section 2. The purpose was to assess the attitude of the participants towards over-the-counter medication and herbal safety and the level of knowledge. Past and current medication use (Q11-Q12) was captured in Section 3. The aim of this section was to determine whether the participants were using any OTC medication or vitamins and whether they had suffered side effects. Current health status of the pregnant women (Q13-Q14) was solicited in Section 4. This was done to establish whether the participants had any chronic diseases that would encourage them to seek medications or smoking that may alter or interfere with, if any, over-the-counter medications they would be using. Section 5 consisted of information on previous children (Q15) born. This section established whether previous children were born with special needs or not. Assessment of over-the-counter drug use (Q16–Q19) was part of Section 6. The aim of this section was to determine the overall knowledge of participants of over-the-counter drugs and whether they intended to increase their knowledge of the OTC drugs they used by reading the accompanying leaflet.

At the completion of the study and as a special service, 31 participants who consented and agreed were provided with information about OTC drugs in the form of short paragraphs via the mobile application WhatsApp. This information was available in both English and Arabic and was provided to the consenting patients in accordance with their preferred language.

### 2.5. Validity and Reliability Testing

Face validity of the questionnaire was determined using the following approach. Despite the fact that the survey questions were taken from a validated study [15], it was sent to two faculty members and one physician to assess the face validity; this is because the original validity questionnaire was subjected to some modifications. Additionally, the survey was provided to four nonparticipants and they were asked to provide feedback.

To assess test-retest reliability, the questionnaire was sent on two separate occasions to ten participants randomly selected from the clinics and hospital. The second follow-up response was obtained two weeks later. Test-retest reliability was calculated using Spearman's correlation coefficient (*r*). The rho value was found to be 0.87, which implies an acceptable level of test-retest reliability.

### 2.6. Data Analysis

The participants' responses were encoded and the data were analyzed using Statistical Package for the Social Sciences (IBM SPSS Statistics for Windows, version 20.0, IBM Corp., Armonk, NY, USA). Descriptive analysis was used to calculate the response proportion of each group of respondents for each item in the questionnaire. Public responses options to the survey questions related to education level, use of over-the-counter drugs, medical history, and over-the-counter drug and vitamin use were reduced to three categories: yes, no, and sometimes. This enabled more reader comprehensible confidence intervals for the relative proportions to be calculated. The level *P* < 0.05 was considered as the cut-off value for statistical significance.

## 3. Results

A total of 140 questionnaires were distributed over the study period of 3 months (October 2016 to December 2016). All of them were returned fully complete, giving a response rate of 100%.

### 3.1. Demographics

Among the 140 pregnant females who participated in this study, the majority were married (137; 97.1%), Arab (nonlocal) (80; 57.1%), and between 21 and 35 years of age (115; 82.1%). Eighty-nine (65.7%) of the participants held a university degree and only 12 (8.6%) reported that they work as healthcare personnel. Details about participants' personal information are presented in Table 1.

### 3.2. Education Level, Use of Over-the-Counter Medication, and Medical History

When the participants were asked if they knew the term “over-the-counter medications,” more than three-quarters (106; 75.7%) of the participants reported “yes” while 34 (24.3%) reported that they had no idea about the meaning of the term. They were then asked based on the previous question about their level of knowledge towards over-the-counter medications (low to medium to advanced); 36 (25.7%) of the participants believe that their knowledge about OTC drug use and safety was low. 81 (57.9%) and 23 (16.4%) of the participants reported their knowledge as medium and advanced levels, respectively. However, the majority (132; 94.3%) of the participants agreed with the survey statement that OTC drugs were not safe to be used during pregnancy. Even though pregnant women included in this study were not happy to use over-the-counter medication during pregnancy, 60 (42.9%) believed in the safety of herbal products during pregnancy.

Of the 140 women included in the study, 137 (97%) reported that they are nonsmokers, 3 (2.1%) were diabetics, 4 (2.9%) were hypertensive patients, and half of them (70; 50%) used at least one over-the-counter medication before their conception day. Participants were divided further; 40 (28.6%) participants reported “yes” to the survey question “Have you ever used an over-the-counter drug or vitamin in your current pregnancy?” Even though majority (129; 92.1%) of the sample pool did not experience any side effects from the drugs they used during their pregnancy, 11 (7.8%) reported some side effects. Constipation (6; 4.3%) and headache (5; 3.6%) were the most common side effects reported by the participants. The response from participants about the use and reading of the medication leaflet was positive and promising. Ninety-eight (70%) of the pregnant females under investigation reported “yes” to the survey question “Do you read/check the accompanying leaflet?” Table 2 summarizes the education level towards over-the-counter drugs, their use, and medical history.

### 3.3. Over-the-Counter Medication Use and Vitamin Use by Pregnant Women

The participants' answers were analyzed and a variety of responses were recorded for the questions concerning the vitamins that the pregnant women took during their pregnancy. They were encouraged to select more than one answer. Folic acid was used by 100 (36.2%) participants, calcium by 79 (28.6%), and iron by 97 (35.1%). The participants were then asked to write down any other vitamins that they were taking. The following question asked them to choose any of the mentioned over-the-counter drugs used during their pregnancy; Panadol was used by more than half of the participants (43; 55.1%) followed by Panadol All in One, (Cold and Flu) (15; 19.2%), Prospan (ivy leaf extract) (11; 14.1%), and ibuprofen (8; 10.3%).  Table 3 summarizes the vitamins and over-the-counter drugs used by the pregnant women.

Responses were recorded for the survey question “Do you know the critical time for the use of over-the-counter drugs during pregnancy?” More than half (73; 55.7%) indicated the first trimester, while 46 (32.9%) believed it depended on the drug. The rest decided on the second trimester (6; 4.3%) and third trimester (10; 7.1%).

## 4. Discussion

In UAE, healthcare, like many other gulf countries, is provided to all residents through primary healthcare centers. Patients gain access to secondary or tertiary care through referral from primary healthcare centers. Both local and insured patients can seek medical services and collect their prescription medications from a wide range of hospital pharmacies free of charge. However, outside the secondary care sector, a major portion of noninsured and expatriate patients obtain their medication from the growing number of private community pharmacies.

It has been reported that, in UAE, like the rest of the world, people tend to go for self-medication for many reasons, which include the high cost of medical consultations, lack of time, long hours of waiting at the physician's clinic, and lack of trust in the physicians' medical knowledge. Furthermore, previous experience with a medical condition and its drug management and the lack or the unavailability of nearby health facilities were identified as reasons for seeking OTC medications [5]. OTC medications are beneficial for the treatment of minor ailments only if there is sufficient knowledge about the correct use of the medicines. There are at least five pieces of information required for appropriate OTC medications use: information about the active ingredient, indication, dosage and administration, side effects, and contraindications. Even though the majority (94.3%) of respondents of this study believed that not all OTC drugs are safe to be used during pregnancy, more than one-quarter (28.6%) of pregnant women respondents reported that OTC medications and other herbal supplements were used during their pregnancy.

This trend is similar to the use reported in Texas (23.0%) and Pakistan (37.9%) [13, 15] and is far less than the usage among women in USA [16]. More than three-quarters (82.0%) of women in USA reported that they have used OTC drugs in the previous six months [16, 17]. This is important if one considers that the level of knowledge that mothers had about medicine was considered to be inadequate to support safe and effective self-medication reported elsewhere [18]. In this study, more than half (57.1%) of the participants disagreed and 42.9% agreed on the survey statement “herbal products are safe in pregnancy.” Such finding is similar to those studies that reported high usage of herbal products among pregnant women [14, 16, 19]. An interesting aspect to consider in this question is the cultural background of the patients answering the question. Even though cultural backgrounds were not asked in this survey, many pregnant women originating from countries like Pakistan or the Indian subcontinent utilized traditional medicines as part of their cultural heritage [20]. The ingredients of these traditional medicines are mostly uncontrolled and used as per traditional practices.

Drug use during pregnancy is common [21]. Commonly used OTC medications are analgesics, antipyretics, cough syrups, antiemetics, herbal products, and nutritional supplements. The incidence of prescribed drugs ranges from 40% to 93% [22, 23] in economically developed countries; variation in this range is explained by exclusion, or respective inclusion of vitamins. Even though not all the drugs are associated with medical complications to the fetus and mother, some may lead to severe damage to both fetus and mother. High doses of acetylsalicylic acid result in increased perinatal mortality, neonatal hemorrhage, low birth weight, prolonged gestation/labor, and possible birth defects. On the other hand, the use of folic acid is essential in the first trimester of pregnancy to prevent neural tube defects [24]. In this study, the most commonly used supplements were folic acid (36.2%), followed by iron (35.1%) and then calcium (28.6%). These drugs are commonly recommended by doctors and are recommended for use during pregnancy.

Our study findings were similar to the study findings of Inamdar et al. [25] and Hanafy et al. [26]; these authors found that 39% of pregnant women used iron and 26.6% used calcium. However, the use of folic acid was higher than that reported in our study. The use of folic acid was 69% in India [25] and 51% in Egypt [26]. Calcium has the benefits of reducing preeclampsia and hypertension and maintaining a healthy heart for both fetus and mother [27]. Iron is known to fight depression and build resistance to stress and disease in the mother as well as being an important part of the red blood cells as mothers have an increased maternal red blood mass [28]. Low iron levels are associated with premature delivery, low birth weight, and infant mortality [29, 30]. Hence, it may be advised for healthcare professionals to advise pregnant women on the proper usage of these medications. Encouraging pharmacists, who are able to counsel patients more commonly, to talk to pregnant women upon the purchase of these products may be a beneficial idea.

The first trimester is an important period of pregnancy; more care should be taken as it is the period of organogenesis, and drug intake during this period has a profound effect on the fetus. Even though more than half (55.7%) of the respondents are aware that the first trimester remains the most critical time for the pregnant women to take any medication as it interferes with the development of the fetus, still 4.3% and 7.1% of the respondents believe that the second and third trimesters are more important than the first trimester.

Analgesic drug use is high, with more than half (55.1%) of the pregnant women using paracetamol during the study period. This is consistent with the findings from Pakistan (43.6%) [13] and Ethiopia (37.5%) [31]. It is common knowledge that every drug incurs a side effect within the human body which is consistent with the finding that paracetamol might have an adverse effect on neurological development (psychomotor, behavioral, and temperamental outcomes for the child), irrelevant of the trimester exposure that took place [32].

Interestingly, 70% of the pregnant females under investigation reported “yes” to the survey statement “read the accompanying leaflet content before using the OTC drugs”. One can presume that one of the reasons behind reading the OTC medication leaflet before taking the OTC was either a lack of knowledge on how to take the drug or fear of side effects of the medication.

*Limitations of the Study.* Even though study participants were asked about which trimester was critical for OTC drug use during pregnancy (trimester timeline), it was not taken into consideration when participants were invited to be a part of the study. This is hence a limitation of this study and it is recommended to consider this for future studies. Secondly, the study was conducted over a period of only three months and patients who were available at the clinics/hospitals were considered for inclusion. The limited sample size and relatively short study period may not allow generalizing the research outcomes. Lastly, patient records are not available to pharmacists in UAE, and pregnant women displayed unwillingness to share personal information with researchers due to cultural sensitivities. This is an important limitation that may be experienced by other researchers within the Arab world as well.

## 5. Conclusion

The reported 40% usage of OTC medications among pregnant women in this study is high and not healthy. The perception of the safety of herbal drugs among the pregnant women is worrisome. There is a need to educate, counsel, and increase awareness among pregnant women regarding safe OTC drug and herbal medicine use while pregnant in Sharjah, UAE.

## Figures and Tables

**Table 1:** Demographic characteristics of the participants.

Characteristic	Frequency (%)
*Nationality*	
Local	42 (30%)
Arab (nonlocal)	80 (57.1%)
Non-Arab (nonlocal)	18 (12.9%)

*Age (years)*	
15–20	3 (2.1%)
21–25	35 (25%)
26–30	51 (36.4%)
31–35	29 (20.7%)
36–40	14 (10%)
41–55	8 (5.7%)

*Marital status*	
Married	137 (97.1%)
Divorced	2 (1.4%)
Widowed	0
Separated	1 (0.7%)

*Educational level*	
Primary/secondary school	10 (7.1%)
High school	35 (25%)
University or college	89 (63.6%)
Other	6 (4.3%)

*Do you have previous children?*	
None	40 (28.6%)
One	43 (30.7%)
Two	24 (17.1%)
More than two	33 (23.6%)

*Occupation*	
Student	9 (6.4%)
Housewife	92 (65.7%)
Healthcare personnel (physician, nurse, or pharmacist)	12 (8.6%)
Employed in the non-healthcare sector	23 (16.4%)
Other	4 (2.9%)

*Do you smoke?*	
Yes	3 (2.1%)
No	137 (97.9%)

**Table 2:** Public responses to the survey questions related to education level, use of over-the-counter drugs, and medical history.

Survey items	Yes *n* (%)	No *n* (%)	Sometimes *n* (%)	95% CI for single proportion for “yes”
Do you know what is meant by over-the-counter drugs?	106 (75.7%)	34 (24.3%)	0	68.6–82.8

Do you think that all over-the-counter drugs are safe to be taken during pregnancy?	8 (5.7%)	132 (94.3%)	0	1.9–9.5

Do you think that all herbal medications are safe to be taken during pregnancy?	60 (42.9%)	80 (57.1%)	0	34.7–51.1

Have you ever used an over-the-counter drug before pregnancy?	70 (50%)	70 (50%)	0	41.7–58.2

Do you read/check the accompanying leaflet?	98 (70%)	11 (7.9%)	31 (22.1)	62.5–77.5

Have you ever used an over-the-counter drug or vitamin in your current pregnancy?	40 (28.6%)	99 (70.7%)	0	21.1–36.1

Did you experience any side effects from the over-the-counter drugs?	11 (7.8%)	129 (92.1%)	0	3.4–12.3
Headache	5 (3.6%)	135 (96.4%)		
Constipation	6 (4.3%)	134 (95/7%)		

Do you have any chronic diseases (hypertension, diabetes, or asthma)?	9 (6.4%)	131 (93.6%)	0	2.4–10.5
Diabetes	3 (2.1%)	137 (97.9%)		
Disc and migraine	1 (0.7%)	139 (99.3%)		
Hypertension	4 (2.9%)	136 (97.1%)		

Do you have a child with special needs? Hereditary disease	1 (0.7%)	139 (99.3%)	0	1.0–2.1

**Table 3:** Public responses on the survey questions related to over-the-counter drug and vitamin use.

Survey items (multiple responses)	Yes *n* (%)	No *n* (%)	95% CI for single proportion for “yes” answer option (%)
Vitamins used by pregnant women			
Folic acid	100 (36.2%)	0	63.9–78.8
Calcium	79 (28.6%)	0	48.3–64.6
Iron	97 (35.1%)	0	61.7–76.9
*Others*	12 (8.4)	0	3.9–13.2
Magnesium	2 (1.4%)	0	
Multivitamins	3 (2.1%)	0	

Medications used by pregnant women			
Panadol	43 (55.1%)		23.1–38.3
Ibuprofen	8 (10.3%)		1.9–9.5
Panadol All in One (Cold and Flu)	15 (19.2%)		10.5–27.9
Prospan or any cough syrup	11 (14.1%)		6.4–21.8

## Appendix

### Awareness and Use of Over-the-Counter Drugs in Pregnant Women

*Tick only one answer*

*Nationality:*
  □ Local
  □ Nonlocal (Arab)
  □ Non-Arab
*Age (years)*
  □ 15–20
  □ 21–25
  □ 26–30
  □ 31–35
  □ 36–40
  □ 41–55
*Marital status:*
  □ Single
  □ Married
  □ Divorced
  □ Widowed
  □ Separated
*Highest education completed*
  □ Primary/secondary school (8-9 years of education)
  □ High school (11–13 years of education)
  □ University or college
  □ Other education; please specify: ……………………
*Work situation at the start of pregnancy*
  □ Student
  □ Housewife
  □ Healthcare personnel (physician, nurse, or pharmacist)
  □ Employed in the non-healthcare sector
  □ Unknown
*Previous children*
  □ None
  □ One
  □ Two
  □ More than two
*Do you know what is meant by over-the-counter drugs?*
  □ Yes
  □ No
*What is your level of knowledge towards over-the-counter drugs?*
  □ Low
  □ Medium
  □ Advanced
*Do you think that all the OTC drugs are safe to be taken during pregnancy?*
  □ Yes
  □ No
*Do you think natural remedies are safe to be taken during pregnancy?*
  □ Yes
  □ No
*Have you ever used an over-the-counter Drug before pregnancy?*
  □ Yes
  □ No
*Have you ever used an OTC drug including vitamins in your current pregnancy?*
  □ Yes
  □ No
  If your answer is yes, please answer the following questions:
(i) Name the OTC drug or vitamin that you are using ………………………
(ii) Have you ever experienced any side effects from the OTC drugs?
  □ Yes
  □ No
(iii) If the answer is yes, answer the question below: after taking the OTC drug or vitamins, have you experienced any of these side effects?
  □ Headache
  □ Cough
  □ Constipation
  □ Common cold
*Do you have any chronic diseases (hypertension, diabetes, asthma, etc.)?*
  □ Yes
  □ No
  If yes, please specify ………………………
*Do you smoke?*
  □ Yes
  □ No
*Do you have a child with special needs?*
  □ Yes
  □ No
  If yes, is it
  □ hereditary disease
  □ for the first time in your family
  □ due to a side effect of an OTC drug, name of the drug …………………………
*Do you know the critical time for OTC drug use during pregnancy?*
  □ First trimester
  □ Second trimester
  □ Third trimester
  □ It depends on the drug
*Do you use any of these vitamins (you can choose more than one answer)?*
  □ Folic acid
  □ Calcium
  □ Iron
  □ Other; please specify: ……………………
*Do you use any of these medications (you can choose more than one answer)?*
  □ Paracetamol
  □ Ibuprofen
  □ Any pain killer
  □ Panadol All in One (Cold and Flu)
  □ Prospan or any cough syrup
*Do you read/check the accompanying leaflet content?*
  □ Yes
  □ No
  □ Sometimes

## References

[1] Matt D. W., Borzelleca J. F.

[2] Mitchell A. A., Gilboa S. M., Werler M. M. (1976). Medication use during pregnancy, with particular focus on prescription drugs. *American Journal of Obstetrics and Gynecology*.

[3] Brunton L. L., Chabner B. A., Knollmann B. C. (2011). *Goodman and Gillmans Pharmacological Basis of Therapeutics*.

[4] Blehar M. C., Spong C., Grady C., Goldkind S. F., Sahin L., Clayton J. A. (2013). Enrolling pregnant women: issues in clinical research. *Women's Health Issues*.

[5] Servey J., Chang J. (2014). Over-the-counter medications in pregnancy. *American Family Physician*.

[6] CDC. Use of Medication in Pregnancy 2017. Available from: https://www.cdc.gov/pregnancy/meds/treatingfortwo/data.html

[7] U.A.E. Population. United Arab Emirates Population 2017 2017. Available from: http://worldpopulationreview.com/countries/united-arab-emirates-population/

[8] U. A. Emirates. Best Countries for Business: Forbes; 2017. Available from: https://www.forbes.com/places/united-arab-emirates/

[9] Kamali F. A., Bastaki H. A. http://www.thenational.ae/lifestyle/any-poor-emiratis-out-there.

[10] Sharjahmedia. About Sharjah 2017. Available from: http://sharjahmedia.ae/en/about-sharjah/history.aspx

[11] Mundi I. http://www.indexmundi.com/g/g.aspx?c=tc&amp;v=25.

[12] Tradingeconomics. United Arab Emirates - Birth rate, crude 2015. Available from: https://tradingeconomics.com/united-arab-emirates/birth-rate-crude-per-1-000-people-wb-data.html

[13] Bohio R., Brohi Z. P., Bohio F. (2016). Utilization of over the counter medication among pregnant women; a cross-sectional study conducted at Isra University Hospital, Hyderabad. *Journal of the Pakistan Medical Association*.

[14] Sawalha A. F. (2007). Consumption of prescription and non-prescription medications by pregnant women: a cross sectional study in palestine. *The Islamic University Journal*.

[15] Chpa.org. Statistics on OTC Use: Chpa.org.; 2016. Available from: http://www.chpa.org/marketstats.aspx

[16] Hong S. H., Spadaro D., West D., Tak S. H. (2005). Patient valuation of pharmacist services for self care with OTC medications. *Journal of Clinical Pharmacy and Therapeutics*.

[17] Kline K. L., Westberg S. M. (2011). Over-the-counter medication use, perceived safety, and decision-making behaviors in pregnant women. *Innovation in Pharmacy*.

[18] Suryawati S. (2003). CBIA: improving the quality of self-medication through mothers active learning. *Essential Drugs Monitor*.

[19] Staff M. C. http://www.mayoclinic.org/healthy-lifestyle/nutrition-and-healthy-eating/in-depth/herbal-supplements/art-20046714.

[20] Hussain S., Malik F., Khalid N. (2010). *Alternative and Traditional Medicines Systems in Pakistan: History, Regulation, Trends, Usefulness, Challenges, Prospects and Limitations*.

[21] van Hasselt J. G. C., Andrew M. A., Hebert M. F., Tarning J., Vicini P., Mattison D. R. (2012). The status of pharmacometrics in pregnancy: highlights from the 3rd American conference on pharmacometrics. *British Journal of Clinical Pharmacology*.

[22] Donati S., Baglio G., Spinelli A., Grandolfo M. E. (2000). Drug use in pregnancy among Italian women. *European Journal of Clinical Pharmacology*.

[23] Nordeng H., Jacobsen G., Nesheim B., Eskild A. (2009). Drug use in pregnancy among parous Scandinavian women. *Norsk Epidemiologi*.

[24] Board B. M. A. https://www.babycentre.co.uk/a561818/buying-pregnancy-multivitamins.

[25] Inamdar I. F., Aswa N. R., Snokar V. K. (2012). Drug utilization pattern during pregnancy. *Indian Medical Gazette*.

[26] Hanafy A. S., Sallam A. S., Kharboush F. I. (2016). Drug utilization pattern during pregna1ncy in Alexandria, Egypt. *European Journal of Pharmaceutical and Medical Research*.

[27] WHO.int. WHO Guideline: Calcium supplementation in pregnant women: WHO.int. ; 2013. Available from: http://apps.who.int/iris/bitstream/10665/85120/1/9789241505376_eng.pdf 24006556

[28] Clevelandclinic.org. Cleveland clinic Clevelandclinic.org.; 2015. Available from: http://my.clevelandclinic.org/

[29] C. s. F. Guide. Folic acid, iron and pregnancy: Government of Canada 2016. Available from: https://www.canada.ca/en/public-health/services/pregnancy/folic-acid-iron-pregnancy.html

[30] Centrum.ca. Are You Getting Enough Iron?: WebMD; 2016. Available from: http://www.webmd.com/baby/are-you-getting-enough-iron

[31] Kassaw C., Wabe N. T. (2012). Pregnant women and non-steroidal anti-inflammatory drugs: Knowledge, perception and drug consumption pattern during pregnancy in Ethiopia. *North American Journal of Medical Sciences*.

[32] Brandlistuen R. E., Ystrom E., Nulman I., Koren G., Nordeng H. (2013). Prenatal paracetamol exposure and child neurodevelopment: A sibling-controlled cohort study. *International Journal of Epidemiology*.

